# Population Structure, Antibiotic Resistance, and Uropathogenicity of Klebsiella variicola

**DOI:** 10.1128/mBio.02481-18

**Published:** 2018-12-18

**Authors:** Robert F. Potter, William Lainhart, Joy Twentyman, Meghan A. Wallace, Bin Wang, Carey-Ann D. Burnham, David A. Rosen, Gautam Dantas

**Affiliations:** a The Edison Family Center for Genome Sciences and Systems Biology, Washington University in St. Louis School of Medicine, St. Louis, Missouri, USA; b Department of Pathology and Immunology, Washington University in St. Louis School of Medicine, St. Louis, Missouri, USA; c Department of Pediatrics, Washington University in St. Louis School of Medicine, St. Louis, Missouri, USA; d Department of Molecular Microbiology, Washington University in St. Louis School of Medicine, St. Louis, Missouri, USA; e Department of Biomedical Engineering, Washington University in St. Louis, St. Louis, Missouri, USA; The Sanger Institute; Houston Methodist Research Institute; Johns Hopkins

**Keywords:** emerging pathogens, *Klebsiella*, antibiotic resistance, microbial genomics, urinary tract infection

## Abstract

Klebsiella variicola is a member of the *Klebsiella* genus and often misidentified as Klebsiella pneumoniae or Klebsiella quasipneumoniae. The importance of K. pneumoniae human infections has been known; however, a dearth of relative knowledge exists for K. variicola. Despite its growing clinical importance, comprehensive analyses of K. variicola population structure and mechanistic investigations of virulence factors and antibiotic resistance genes have not yet been performed. To address this, we utilized *in silico*, *in vitro*, and *in vivo* methods to study a cohort of K. variicola isolates and genomes. We found that the K. variicola population structure has two distant lineages composed of two and 143 genomes, respectively. Ten of 145 K. variicola genomes harbored carbapenem resistance genes, and 6/145 contained complete virulence operons. While the β-lactam *bla*_LEN_ and quinolone *oqxAB* antibiotic resistance genes were generally conserved within our institutional cohort, unexpectedly 11 isolates were nonresistant to the β-lactam ampicillin and only one isolate was nonsusceptible to the quinolone ciprofloxacin. K. variicola isolates have variation in ability to cause urinary tract infections in a newly developed murine model, but importantly a strain had statistically significant higher bladder CFU than the model uropathogenic K. pneumoniae strain TOP52. Type 1 pilus and genomic identification of altered *fim* operon structure were associated with differences in bladder CFU for the tested strains. Nine newly reported types of pilus genes were discovered in the K. variicola pan-genome, including the first identified P-pilus in *Klebsiella* spp.

## Abstract (precis)

Infections caused by antibiotic-resistant bacterial pathogens are a growing public health threat. Understanding of pathogen relatedness and biology is imperative for tracking outbreaks and developing therapeutics. Here, we detail the phylogenetic structure of 145 K. variicola genomes from different continents. Our results have important clinical ramifications as high-risk antibiotic resistance genes are present in K. variicola genomes from a variety of geographic locations and as we demonstrate that K. variicola clinical isolates can establish higher bladder titers than K. pneumoniae. Differential presence of these pilus genes inK. variicola isolates may indicate adaption for specific environmental niches. Therefore, due to the potential of multidrug resistance and pathogenic efficacy, identification of K. variicola and K. pneumoniae to a species level should be performed to optimally improve patient outcomes during infection. This work provides a foundation for our improved understanding of K. variicola biology and pathogenesis.

## INTRODUCTION

Klebsiella variicola was initially believed to be a plant-associated, distant lineage of Klebsiella pneumoniae; however, it has subsequently been recovered from human clinical specimens (1). Despite increasing knowledge on the distinctness of K. variicola, K. pneumoniae, and Klebsiella quasipneumoniae, misidentification within the clinical microbiology lab commonly occurs (2, 3). This may have clinical implications, as one study demonstrated that K. variicola-infected patients have higher mortality than K. pneumoniae-infected patients (4). Furthermore, several virulence genes (VGs), including siderophores, allantoin utilization genes, and glycerate pathway genes, have been reported in select K. variicola strains (5, 6). K. variicola has been shown to contain a large pan-genome that is distinct from K. quasipneumoniae and K. pneumoniae, but the functional consequences of differential gene content have not been explored (2, 7).

In this study, we retrospectively analyzed a cohort of *Klebsiella* isolates collected from 2016 to 2017 at Washington University in St. Louis School of Medicine/Barnes-Jewish Hospital Clinical Microbiology Laboratory (WUSM) for possible K. variicola strains using matrix-assisted laser desorption ionization–time of flight mass spectrometry (MALDI-TOF MS) and *yggE* PCR-restriction fragment length polymorphism (RFLP) assays. We performed Illumina whole-genome sequencing (WGS) to compare K. variicola from our institution with publicly available genomes in the first global evaluation of this species. We particularly focused on annotation of canonical *Klebsiella* VGs and antibiotic resistance genes (ARGs) and then assessed their functional consequences using *in vitro* assays and *in vivo* murine infections. Our results demonstrate that population structure, antibiotic resistance, and uropathogenicity of K. variicola are generally similar to K. pneumoniae, but variability among K. variicola genomes has important clinical implications with various strain efficacies in a murine model of urinary tract infection (UTI).

## RESULTS

### Average nucleotide identity and MALDI-TOF MS can differentiate K. variicola from K. pneumoniae.

**FIG 1:**
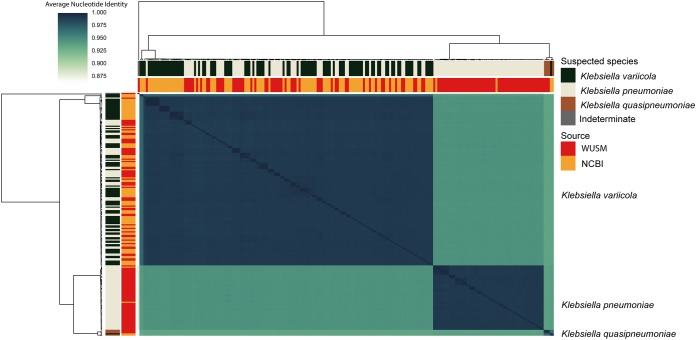
Pairwise average nucleotide identity cluster map of WUSM and NCBI *Klebsiella.* Hierarchical clustering and heat map of pairwise ANIm values among all isolates. The source of isolates (WUSM or NCBI) and initial species delineation (K. variicola, K. pneumoniae, or K. quasipneumoniae) are shown as colored bars adjacent to the heat map. The three major blocks are labeled by their final species determination.

We performed Illumina WGS on 113 isolates that are commonly misidentified as K. pneumoniae (K. variicola [*n* = 56], K. quasipneumoniae [*n* = 3], K. pneumoniae [*n* = 53], and Citrobacter freundii [*n* = 1]). They were identified by Bruker Biotyper MALDI-TOF MS and *yggE* RFLP assays from a variety of adult infection sites (see Table S1 in the supplemental material). The isolates were retrieved from the Barnes-Jewish Hospital clinical microbiology laboratory (St. Louis, MO, USA) in 2016 to 2017. We used pyANI with the mummer method to calculate the pairwise average nucleotide identity (ANIm) between the isolates in our cohort and retrieved publicly available *Klebsiella* genomes (*n* = 90) (8, 9) (Table S1). The C. freundii isolate was originally classified as K. pneumoniae from the Vitek MS MALDI-TOF MS v2.3.3 but was later determined to be Citrobacter freundii by Bruker Biotyper MALDI-TOF MS. The *yggE* PCR-RFLP was indeterminate for this isolate. Confirmatory *yggE* PCR-RFLP had 94.6% (53/56) concordance with MALDI-TOF for prediction of K. variicola within our cohort (Fig. 1). While one genome was dropped from downstream analysis, the other 55 WUSM K. variicola genomes all had >95% ANIm with the reference genome of K. variicola At-22 (5). K. variicola HKUPOLA (GCA_001278905.1) had >95% ANIm with K. quasipneumoniae ATCC 7000603 reference genome but not K. variicola At-22, indicating that it is likely a misannotated K. quasipneumoniae isolate and not a K. variicola isolate. The remainder of the NCBI K. variicola genomes clustered with K. variicola At-22 and the WUSM K. variicola cohort. One hundred percent (41/41) of the K. pneumoniae genomes from NCBI that were suspected to be K. variicola due to BLAST similarity had >95% ANIm with K. variicola At-22 but not K. pneumoniae HS11286 or K. pneumoniae CAV1042 (Fig. 1).

10.1128/mBio.02481-18.5 TABLE S1 Clinical characteristics and sequence statistics of isolates sequenced in this investigation. Publicly available genomes used in this study. ANIb values from JSpecies for representative isolates in the two K. variicola lineages. Download Table S1, XLSX file, 0.1 MB. Copyright © 2018 Potter et al. 2018 Potter et al. This content is distributed under the terms of the Creative Commons Attribution 4.0 International license.

Hierarchical clustering of the pairwise ANIm values replicated previous phylogenetic analysis showing that K. pneumoniae and K. quasipneumoniae are more closely related to each other than to K. variicola (Fig. 1). Interestingly, the clustering pattern within K. variicola indicated that two isolates, KvMX2 (FLLH01.1) and YH43 (GCF_001548315.1), are more closely related to one another than to the remainder (143/145) of the K. variicola genomes. Given that K. quasipneumoniae can be differentiated into two subspecies based on ANI with the BLAST method (ANIb), we used the JSpecies ANIb program to specifically compare KvMX2 and YH43 with K. pneumoniae ATCC BAA-1705, K. quasipneumoniae ATCC 7000603, and 3 other K. variicola genomes (10). KvMX2 and Yh43 have 98.02% ANIb with one another but an average of 96.67%, 96.65%, and 96.68% ANIb with WUSM_KV_53, WUSM_KV_15, and K. variicola At-22, respectively (Table S1). Consistent with our pyANI ANIm result, none of the K. variicola strains had >95% ANIb with K. pneumoniae ATCC BAA-1705 or K. quasipneumoniae ATCC 7000603. These data suggest that MALDI-TOF MS or *yggE* PCR-RFLP may be effective means to differentiate K. variicola from K. pneumoniae in the absence of WGS.

### K. variicola population structure has 2 lineages and 26 clusters in the second lineage.

**FIG 2:**
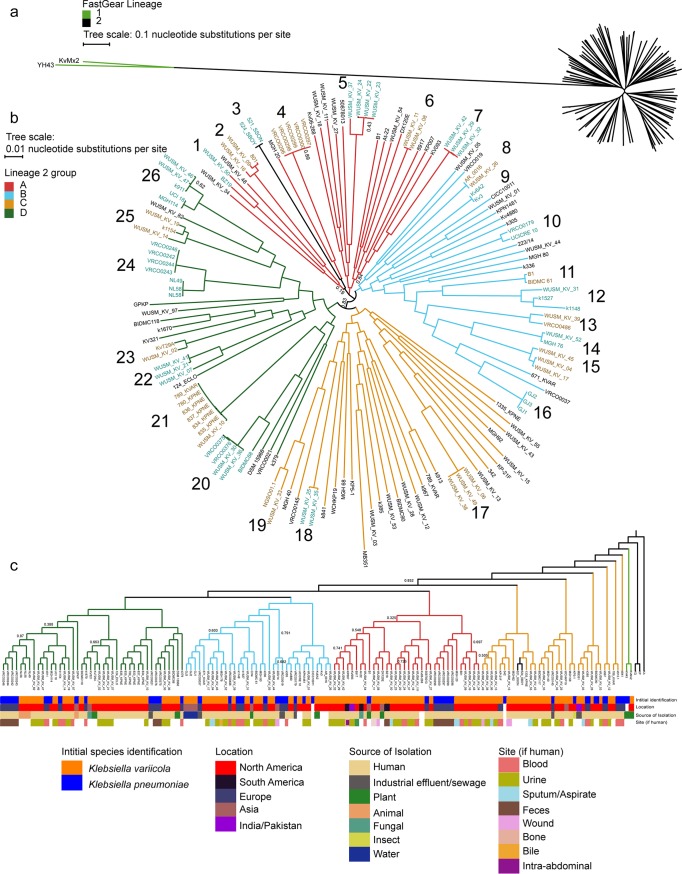
Population structure of K. variicola genomes. (a) Approximate-maximum-likelihood tree of the total 145 K. variicola genomes and annotation of FastGear lineage identification. (b) Recombination-free parSNP tree of the closely related lineage 2 genomes with quantitative clustering from ClusterPicker added as alternating teal and brown labels adjacent to cluster number (1 to 26). Bootstrap support values below 80% are depicted as node labels. (c) Monophyletic groups of these clusters were colored if they were similar in the dendrogram showing the evolutionary context of the cluster compared to K. pneumoniae (KP), K. quasipneumoniae (KQ), and K. aerogenes (KA). Relevant metadata for initial identification, geographic location, source of isolation, and body site are adjacent to the assembly names. Bootstrap support values below 80% are depicted as node labels.

Core-genome alignment of the 1,262 genes at 90% identity shared by strains in all *Klebsiella* species and a Kluyvera georgiana outgroup shows that the K. variicola isolates are in a cluster with K. pneumoniae, K. quasipneumoniae, and the newly described K. quasivariicola (11) (Table S2; Fig. S1). Core-genome alignment of the 3,430 core genes at 95% nucleotide identity for the entire gene length by all 145 K. variicola genomes indicates that KvMX2 and Yh43 are distantly related to the other 143 genomes (Fig. 2a; Table S2). These other genomes form a star-like phylogeny showing deep-branching clusters radiating from the center of the tree. FastGear, which uses hierBAPS to identify lineages and then searches for recombination between lineages, supported the differentiation of KvMX2 and Yh43 into a separate lineage from the other genomes and identified 6 instances of recombination between these two lineages (Table S3) (12, 13).

10.1128/mBio.02481-18.2 FIG S1 FastTree approximate-maximum- likelihood tree of 4 strains in K. variicola and strains in Klebsiella oxytoca, Klebsiella michiganensis, Klebsiella grimontii, Klebsiella aerogenes, Klebsiella quasivariicola, Klebsiella pneumoniae, and Klebsiella quasipneumoniae. Download FIG S1, TIF file, 1.5 MB. Copyright © 2018 Potter et al. 2018 Potter et al. This content is distributed under the terms of the Creative Commons Attribution 4.0 International license.

10.1128/mBio.02481-18.6 TABLE S2 Genes used for core genome alignment of *Klebsiella* species in Fig. S1 in the supplemental material. Genes used for core genome alignment of all 145 K. variicola isolates in Fig. 2a. Genes used for core-genome alignment of the 143 K. variicola lineage 2 isolates in Fig. S2. Genes used for core-genome alignment of the 7 genomes in cluster 21. Genes used for core-genome alignment of the 145 K. variicola isolates and a K. pneumoniae, K. quasipneumoniae, and K. aerogenes outgroup in Fig. 2c. Download Table S2, XLSX file, 0.6 MB. Copyright © 2018 Potter et al. 2018 Potter et al. This content is distributed under the terms of the Creative Commons Attribution 4.0 International license.

10.1128/mBio.02481-18.7 TABLE S3 Analysis of recombination in the two K. variicola lineages by FastGear. Analysis of recombination in the lineage 2 K. variicola genomes by parSNP. Download Table S3, XLSX file, 0.02 MB. Copyright © 2018 Potter et al. 2018 Potter et al. This content is distributed under the terms of the Creative Commons Attribution 4.0 International license.

Phylogenomic network analysis and quantification of recombination from parSNP showed minimal recombination within the 143 K. variicola lineage 2 genomes, with approximately 1.62% of the K. variicola genome believed to be recombinant (Fig. S2a; Table S3) (14). The Nearest Neighbor network of the 3,496 genes shared by the lineage 2 genomes and a recombination-free phylogenetic tree of the 143 genomes from parSNP showed many deep-branching clades with a star-like phylogeny (Fig. 2b; Table S2). This tree topology was similar with and without recombination, which suggests that K. variicola lineages emerged early from a single common ancestor into equally distant clades across different environments (Fig. S2b). Quantitative clustering of the 143 genomes in the second lineage with ClusterPicker showed that 56.6% (81/143) of genomes fall into 26 clusters, with 57.7% (15/26) of the clusters containing more than 2 genomes (Fig. 2b) (15). Only 46.2% (12/26) of clusters contain isolates from both WUSM_KV and NCBI. The largest clusters, 24 and 21, each contain 7 genomes. Cluster 21 contained WUSM_KV_10 and 6 genomes from an analysis of patient isolates at an intensive care unit in Seattle, WA (USA). Although they were in the same cluster, WUSM_KV_10 differed from these isolates at 1,882 sites across the 4,867 genes shared at 95% identity (Table S2 and Table S4).

10.1128/mBio.02481-18.3 FIG S2 (a) Equal angle nearest neighbor phylogenetic network for the 143 K. variicola genomes in lineage 2. (b) FastTree approximate-maximum-likelihood tree with recombinant regions for the closely related lineage 2 genomes. Bootstrap support values below 80% are depicted as node labels. Download FIG S2, TIF file, 3.4 MB. Copyright © 2018 Potter et al. 2018 Potter et al. This content is distributed under the terms of the Creative Commons Attribution 4.0 International license.

10.1128/mBio.02481-18.8 TABLE S4 VCF file output showing SNPs between WUSM_KV_10 and the 6 other genomes in cluster 21. Download Table S4, TXT file, 0.1 MB. Copyright © 2018 Potter et al. 2018 Potter et al. This content is distributed under the terms of the Creative Commons Attribution 4.0 International license.

To better understand the context of the 4 groups in lineage 2, we aligned the 2,932 genes shared among the 145 K. variicola genomes, *Klebsiella* (formerly *Enterobacter*) *aerogenes* KCTC 2190, K. quasipneumoniae ATCC 700603, and K. pneumoniae ATCC BAA-1705 at ≥90% identity to create a dendrogram (Fig. 2c; Table S2). This method preserved the conservation of the lineage 2 groups but showed a different order. The only discrepancy observed is that, in the lineage 2 phylogenetic tree, cluster 3 appeared to be in the A group; however, both 521_SSON and 524_SBOY are more similar to C group genomes in the dendrogram. This incongruence is consistent with cluster 3 radiating away from cluster 4 near the center of the phylogenetic tree (Fig. 2b). Addition of metadata onto the dendrogram showed that the K. variicola cohort spans most geographic locations, with the notable exception of Africa and Oceania (Fig. 2c). The K. variicola genomes showed a remarkable level of source diversity, with representative isolates from animals (*n* = 4), fungi (*n* = 2), plants (*n* = 7), water (*n* = 3), and industrial waste (*n* = 6). However, as a testament to the pathogenic potential of K. variicola, 79.5% (114/145) of genomes came from sites associated with humans. Of the human-associated sites, 40.4% (46/114) came from urine and 19.2% (22/114) came from blood (Fig. 2c). We did not observe any apparent association with geography, habitat, or infection site for any of the K. variicola clades. Sixty-seven of 145 isolates had a sequence type (ST) identified using the K. pneumoniae multilocus sequence type scheme (Table S5). Consistent with the distance between lineages, 44 different STs were identified. ST1562 and ST641 had the highest number of isolates (*n* = 4). In summary, these data demonstrate that K. variicola has a diverse population structure and can be found in a variety of environmental and host niches.

10.1128/mBio.02481-18.9 TABLE S5 Antibiotic resistance genes, virulence genes, and plasmid replicons identified in the 145 K. variicola genomes. Download Table S5, XLSX file, 0.04 MB. Copyright © 2018 Potter et al. 2018 Potter et al. This content is distributed under the terms of the Creative Commons Attribution 4.0 International license.

### Acquired ARGs and VGs are not restricted to any K. variicola cluster.

**FIG 3:**
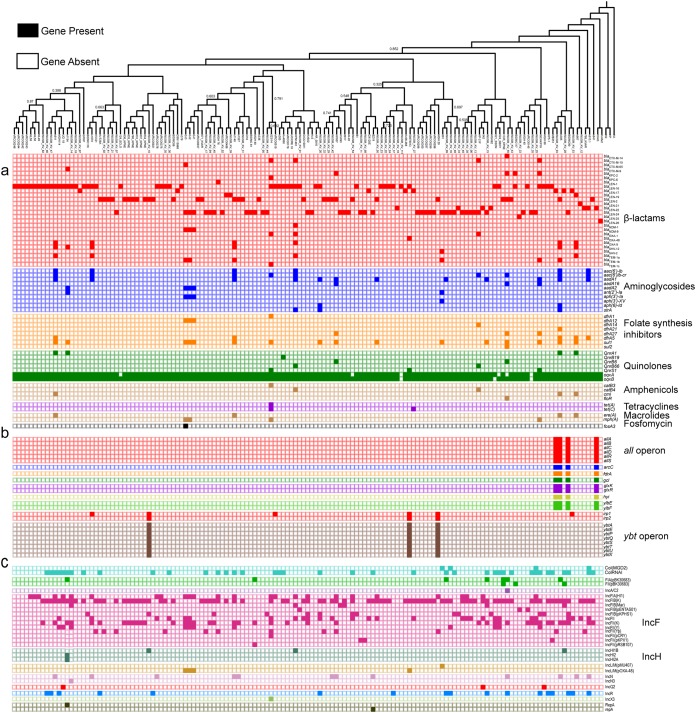
Distribution of acquired antibiotic resistance and virulence genes in the K. variicola cohort. Presence/absence matrix of ARGs (a), virulence genes (b), and plasmid replicons (c) ordered for all K. variicola genomes against the dendrogram from Fig. 2c.

We applied ResFinder to determine the burden of acquired ARGs among the K. variicola strains (Fig. 3a; Table S5) (16). β-Lactamase genes were the most abundant ARG in the K. variicola cohort (*n* = 26). As expected, *bla*_LEN_ was almost universally conserved, as 837_KPNE was the only isolate without one identified. Ten different *bla*_LEN_ alleles were found. *bla*_LEN-16_ was most common (51/145), followed by *bla*_LEN-24_ (40/145) and *bla*_LEN-2_ (31/145). Carbapenemases were rare, but *bla*_KPC-2_ (4/145), *bla*_KPC-6_ (1/145), *bla*_NDM-1_ (1/145), *bla*_NDM-9_ (3/145), and *bla*_OXA-48_ (1/145) were each identified across a total of 10/145 strains. *bla*_CTX_, *bla*_SHV_, *bla*_TEM_, and noncarbapenemase *bla*_OXA_ genes were also identified, but we did not detect any class C β-lactamase genes or non-*bla*_NDM_ class B β-lactamase genes. Aminoglycoside ARGs (*n* = 10), including members of the *aac*, *aad*, *aph*, and *str* families, comprised the second most abundant class. ARGs against folate synthesis inhibitors (*n* = 8), quinolones (*n* = 7), amphenicols (*n* = 4), tetracyclines (*n* = 2), macrolides/lincosamides/streptogramins (*n* = 2), and fosfomycin (*n* = 1) were also found (Fig. 3a). In addition to the near-total conservation of *bla*_LEN_, the quinolone efflux pump components *oqxAB* were found in almost all isolates (139/145). Across the 145 genomes, the median and mode number of ARGs were both 3. A 6.89% (10/145) proportion of genomes harbored ≥10 ARGs, including WUSM_KV_55 from our cohort.

We used the K. pneumoniae BIGSdb database (https://bigsdb.pasteur.fr/klebsiella/klebsiella.html) and BLASTN to identify canonical *Klebsiella* VGs in the *K. variicola* strains (Fig. 3b; Table S5). In contrast to ARGs, previously characterized *Klebsiella* VGs were found only sporadically in the K. variicola cohort. Interestingly, the *all* allantoin utilization operon and *arc*, *fdrA*, *gcl*, *glxKR*, *hyi*, and *ybbWY* genes were found in the distantly related YH43 genome as well as the closely related BIDMC90, k385, and WUSM_KV_03 genomes. *irp12* and the *ybt* operon were found together in the three isolates 50878013, MGH 20, and WUSM_KV_10. *irp1* was found on 3 additional instances but with no other VGs. Among 8 isolates containing the full *all* or *ybt* operon, six had only 3 ARGs; however, 50878013 contains the *ybt* operon and *irp12* and has 5 ARGs, including the *bla*_OXA-48_ carbapenemases, while k385 had 17 ARGs but no carbapenemases.

We used the *Enterobacteriaceae* PlasmidFinder database to identify characterized plasmid replicons in the K. variicola genomes (17). Twenty-nine unique replicons were identified in 11 groups, but 41% (12/29) of replicons were in the IncF group. A single IncF replicon also had the greatest conservation across K. variicola genomes, as 57.9% (84/145) of genomes contained the IncF(K) replicon (GenBank accession no. JN233704). We found a significant association between isolates that harbored greater than the median number of ARGs and greater than the median number of plasmid replicons using the chi-square test (*P* < 0.00001) (Table S5).

### WUSM K. variicola cohort strains are susceptible to most antibiotics.

**FIG 4:**
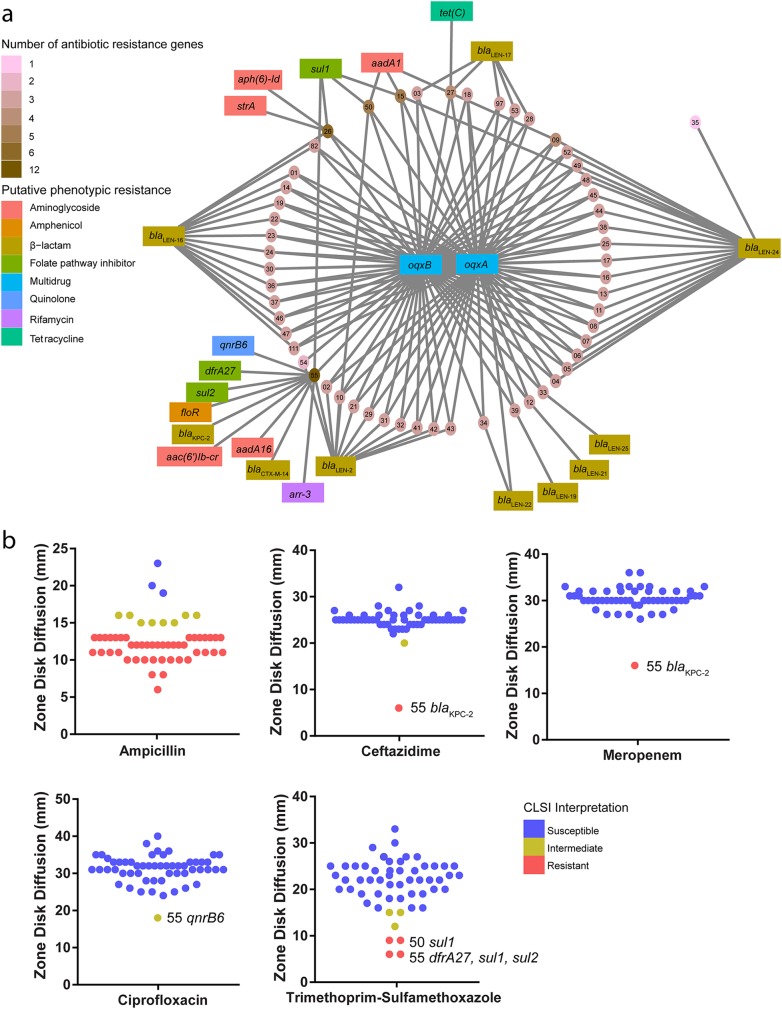
WUSM K. variicola strains have a low burden of ARGs and are generally susceptible to antibiotics. (a) Network diagram depicting each WUSM_KV isolate and ARG as nodes. ARGs are colored in accordance with predicted phenotypic resistance from ResFinder, and WUSM_KV genomes are colored by the burden of ARGs. (b) Scatter plots depicting Kirby-Bauer disk diffusion size (mm) from phenotypic susceptibility testing. Each plot represents an isolate, and the plots are colored according to CLSI interpretation. Those with atypical resistance are listed by name with putative ARGs.

We constructed a network diagram of ARGs and isolates to identify connectivity within the K. variicola strains from our cohort (Fig. 4a). WUSM_KV_55 had twice as many ARGs (*n* = 12) as the next closest isolate, WUSM_KV_26 (*n* = 6). Most notably, WUSM_KV_55 contained the carbapenemase gene *bla*_KPC-2_. In addition to the core β-lactamase *bla*_LEN-2_, this isolate also contained a *bla*_CTX-M-14_ gene. Redundancy was again observed for the ARGs against aminoglycosides and sulfonamides, as WUSM_KV_55 contained *aac(6′)lb-cr*, *aadA16*, *sul1*, and *sul2*. Within our cohort, this isolate was the only isolate found to harbor additional quinolone (*qnrB6*), rifampin (*arr-3*), and amphenicol (*floR*) ARGs. Interestingly, it possesses *oqxB* but not *oqxA*. Conversely, WUSM_KV_35 harbored the lowest number of acquired ARGs, as it lacked *oqxAB* but carried *bla*_LEN-24_.

We used Kirby-Bauer disk diffusion to quantify phenotypic resistance of the WUSM K. variicola strains to several clinically relevant antibiotics (Fig. 4b). *Klebsiella* species are generally considered intrinsically resistant to ampicillin due to a conserved β-lactamase gene. In our cohort, 3/55 isolates were unexpectedly susceptible to ampicillin while the rest were resistant. Despite phenotypic sensitivity to ampicillin, the genomes for WUSM_KV_25, WUSM_KV_34, and WUSM_KV_82 encode *bla*_LEN-24_, *bla*_LEN22_, and *bla*_LEN-16_, respectively. These *bla*_LEN_ alleles were also found in isolates intermediate and resistant to ampicillin. As expected, WUSM_KV_55 was the only isolate resistant to both meropenem and ceftazidime, presumably due to carriage of *bla*_KPC-2_. Additionally, it was the only isolate intermediate to ciprofloxacin. Four isolates were resistant to trimethoprim-sulfamethoxazole, but only WUSM_KV_50 and WUSM_KV_55 had identified ARGs that would explain this phenotype.

Review of a 2017 composite antibiogram from a microbiology laboratory serving 5 hospitals in the St. Louis region (Missouri, USA), based on first isolate per patient per year, revealed that, in general, K. pneumoniae (*n* = 1,522) had decreased susceptibility to all reported antimicrobials compared to K. variicola (*n* = 144), except for meropenem (99% susceptibility for both species). Most notably, K. pneumoniae exhibited decreased susceptibility, compared to K. variicola, with ampicillin-sulbactam (63% versus 93% susceptible), nitrofurantoin (66% versus 86% susceptible), and trimethoprim-sulfamethoxazole (80% versus 90% susceptible).

### Changes in *fim* operon are associated with uropathogenicity in a murine UTI model.

**FIG 5:**
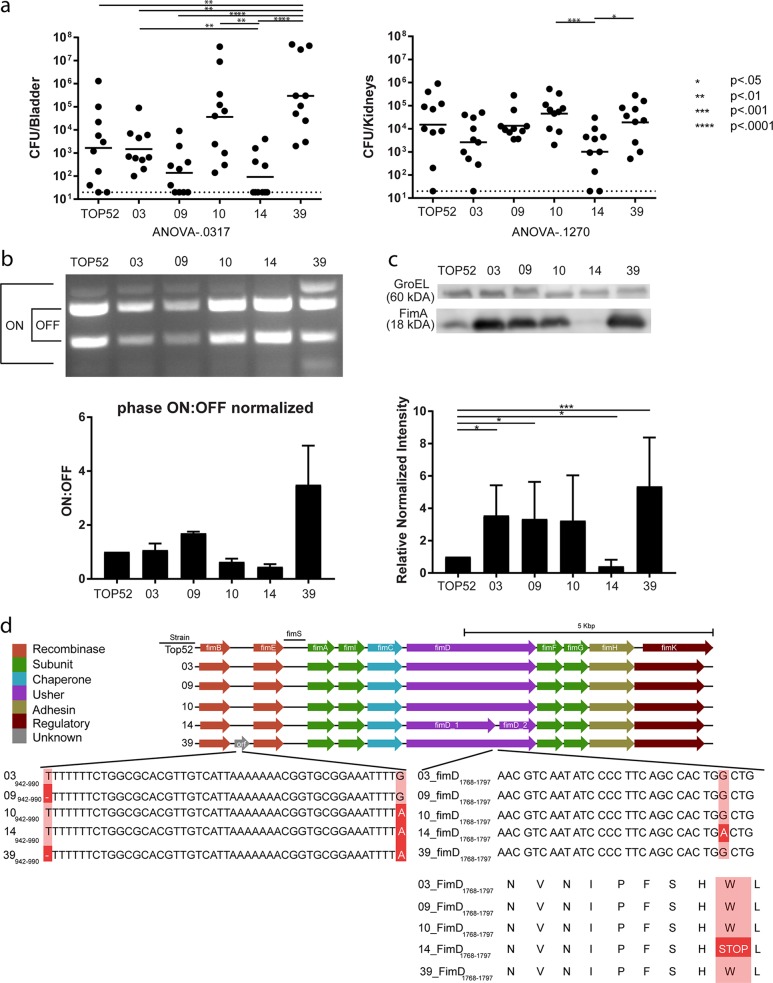
Changes in *fim* operon are associated with outcomes in mouse UTI model. (a) CFU/bladder and CFU/kidney of K. pneumoniae TOP52 and WUSM_KV isolates 24 h after transurethral bladder inoculation of C3H/HeN mice. Short bars represent geometric means of each group, and dotted lines represent limits of detection. (b) *fimS* phase assay and quantification with respective bands indicating the “ON” and “OFF” position labeled. (c) Immunoblot for FimA and GroEL, with quantification shown below. (d) Easyfig illustration of genes in the *fim* operon and Jalview of the nucleotides and amino acids for the *fimB*/*fimE* intergenic region and *fimD* gene.

Given that 70% (39/56) of K. variicola strains from our cohort were isolated from the human urinary tract, we wanted to assess uropathogenicity in a diverse subset of these isolates. We transurethrally inoculated C3H/HeN mice with 10^7^ CFU/ml of 5 individual K. variicola strains, or the model uropathogenic K. pneumoniae TOP52 strain, for comparison (Fig. 5a) (3, 18, 19). Similarly to previously published infections with K. pneumoniae TOP52, the K. variicola strains exhibited large variations in bacterial CFU recovered from the bladder at 24 h postinfection (hpi). Compared to TOP52, WUSM_KV_39 was the only isolate with a significantly increased bladder burden (*P* = 0.0094). Bacterial loads of WUSM_KV_10 and WUSM_KV_39 were both significantly higher than WUSM_KV_09 and WUSM_KV_14 (Fig. 5a). Despite this variability among bladder CFU results, the results of kidney titer determinations at 24 hpi were not significantly different among strains by ANOVA (*P* = 0.1270). As observed in the bladder, however, WUSM_KV_10 and WUSM_KV_39 achieved significantly higher kidney CFU than WUSM_KV_14.

Given the variation in bladder burden, we wanted to assess if differences in uropathogenicity could be related to expression of type 1 pili, a key virulence factor for UTI encoded by the *fim* operon (19, 20). In K. pneumoniae and Escherichia coli, expression of type 1 pili is controlled by a region of invertible DNA (*fimS* site) (20, 21). Orientation of the *fimS* site in the “ON” position enables production of type1 pili and increased urovirulence. Under identical growth conditions, WUSM_KV_39 had a higher population with the *fimS* promoter region in the “ON” orientation than the other strains tested (Fig. 5b). Furthermore, consistent with its success in the bladder, WUSM_KV_39 was found to produce the greatest amount of FimA (the main structural component of type 1 pili), as measured by immunoblotting (Fig. 5c). WUSM_KV_03, WUSM_KV_09, and WUSM_KV_39 all produced significantly more FimA than K. pneumoniae TOP52. Interestingly, WUSM_KV_14 did not produce appreciable levels of FimA by this assay (Fig. 5c).

As we discovered significant variability in type 1 piliation, we specifically investigated changes in *fim* operon sequence between these isolates by viewing the Prokka coding sequence annotation in Easyfig and Jalview (Fig. 5d) (22, 23). We found that WUSM_KV_14 had a predicted truncated FimD usher sequence. A guanine-to-adenine single nucleotide polymorphism (SNP) in the *fimD* gene changed a predicted tryptophan residue into a premature stop codon, likely explaining the observed lack of production of type 1 pili. Additionally, in WUSM_KV_39, Prokka annotated a hypothetical protein in the intergenic region between *fimB* and *fimE* and included a gap replacing a thymine and a guanine-to-adenine SNP. The altered *fimB/fimE* intergenic region in WUSM_KV_39 may play a role in its increased expression of type 1 pili. Together, these data demonstrate that variation exists among K. variicola genomes that may account for differential urinary tract niche proclivity among isolates.

### K. variicola contains both conserved and novel usher genes.

**FIG 6:**
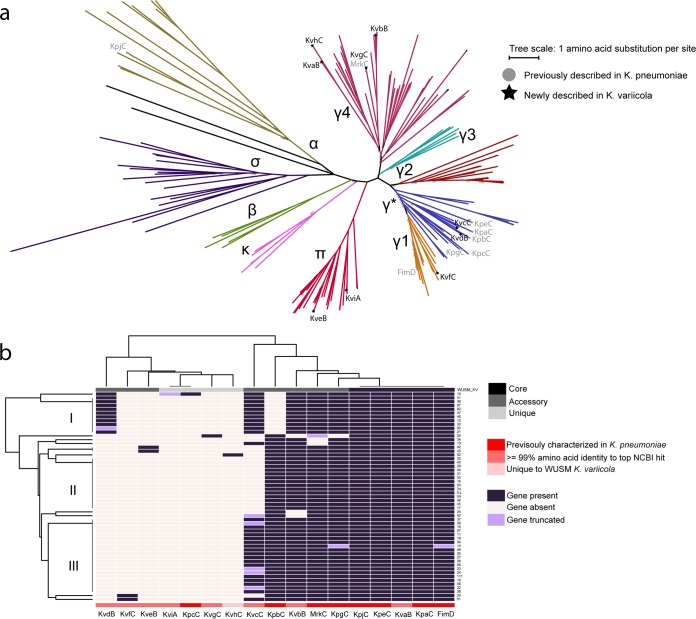
K. variicola carries both conserved and novel usher genes. (a) Approximate-maximum-likelihood tree of the usher amino acid sequences described by Nuccio and Baumler (25) and representatives of the 17 usher sequences identified in the WUSM_KV pan-genome. (b) Hierarchical clustering of the presence/absence matrix of each and annotation of relevant features related to each usher.

The *fim* operon is one of the best-characterized chaperone-usher pathways (CUPs); given the observed importance of the *fim* operon in K. variicola uropathogenicity, we searched the pan-genome of our K. variicola cohort to identify the complete repertoire of CUP operons (24). Seventeen unique usher sequences at 95% identity were identified across the 55 WUSM K. variicola genomes, and an amino acid sequence alignment showed that they were distributed in 5 Nuccio and Baumler (25) clades (Fig. 6a; Table S6). From this analysis, we discovered 9 new usher genes previously undescribed in *Klebsiella*, which we name *kva* through *kvi* (Table S6). KviA and KveB usher sequences were found to cluster within the pi (π) clade, making them the first description of a P-pilus apparatus in *Klebsiella.* The recently named γ* subclade contained the greatest amount (7/17) of K. variicola usher sequences; 5 of these 7 were previously reported in K. pneumoniae, while KvcC and KvdB are first reported here.

10.1128/mBio.02481-18.10 TABLE S6 Usher sequences used by Nuccio and Baumler (2007) for Fig. 6a alignment. Description of newly reported chaperone usher pilus operons identified in K. variicola. Download Table S6, XLSX file, 0.1 MB. Copyright © 2018 Potter et al. 2018 Potter et al. This content is distributed under the terms of the Creative Commons Attribution 4.0 International license.

FimD and the usher sequences for KpaC, KvaB, KpeC, and KpjC were present in all 55 WUSM *K. variicola* isolates (Fig. 6b). KvgC, KvhC, KviA, and KpcC were each found in only one isolate. KpgC, MrkC, KvbC, KpbC, KvcC, KveB, KvfC, and KvdB can be considered accessory usher sequences in this cohort, as they were absent in certain strains. The most notable pattern evident from the hierarchical clustering of the presence/absence for all usher genes in our K. variicola cohort is that isolates WUSM_KV_10 through WUSM_KV_21 all carry the KvdB sequence but not KpbC.

Eight of the 9 newly described usher sequences had highest BLASTP hits of ≥99% identity across the entire length of the gene against the nonredundant protein sequences database in April 2018, and all of them were previously annotated as being found in *Enterobacteriaceae*, *Klebsiella*, or K. variicola (Table S6). All of the usher genes except *kvi* were in operons that included a chaperone, at least one subunit, and a putative adhesin (Table S6). KvhC, the usher protein with the lowest BLASTP identity value, had 76% identity to several genes from *Enterobacter* species (Fig. S3a). The contig with the *kvh* operon also contained several genes that had possible roles in prophage integration and transposase activity (Fig. S3b). Our results indicate that K. variicola strains harbor a diverse set of usher genes, which may augment K. variicola fitness across a variety of environmental niches, and these operons may be acquired from other *Enterobacteriaceae*.

10.1128/mBio.02481-18.4 FIG S3 (a) Alignment of KvhC amino acid sequence with the highest identified BLASTP hits and KvaB. Bootstrap support values below 80% are depicted as node labels. (b) Easyfig image showing the genetic structure of the contig containing the *kvh* operon and genes related to mobilization in WUSM_KV_52. Download FIG S3, TIF file, 1.0 MB. Copyright © 2018 Potter et al. 2018 Potter et al. This content is distributed under the terms of the Creative Commons Attribution 4.0 International license.

## DISCUSSION

A previous phylogenomic study used split-network analysis to demonstrate that the K. variicola phylogroup (formerly KPIII) is distinct from K. pneumoniae (KPI) and K. quasipneumoniae (KPII) (26). As an orthogonal method, we used ANI software, the gold standard for *in silico* species delineation, to recreate this differentiation of phylogroups as separate species (8). Historically, differentiation between K. pneumoniae and K. variicola has been difficult, as evidenced by misannotation of K. variicola as K. pneumoniae in public genome sequence databases (Fig. 1). These misannotated K. variicola strains came from a variety of geographic regions and were not exclusive to any cluster. Within our sequenced cohort, differentiation of K. variicola from K. pneumoniae and K. quasipneumoniae using MALDI-TOF MS and *yggE* PCR-RFLP was supported by ANI. This indicates that *yggE* PCR-RFLP (3) would be a feasible alternative for clinical labs across the globe lacking access to MALDI-TOF MS or WGS. Additionally, hierarchical clustering of the ANI values and core-genome phylogeny demonstrated that 2 K. variicola genomes were distinctly separate from the other 143 in our cohort. ANIb values between these genomes and the other K. variicola genomes were ∼96%, similar to what was observed for K. quasipneumoniae. The differences in ANIb values contributed to the delineation of K. quasipneumoniae into two subspecies, Klebsiellaquasipneumoniae subsp. *quasipneumoniae* and Klebsiellaquasipneumoniae subsp. *similipneumoniae* (27). Further phenotypic comparisons, including the sole carbon source utilization used for differentiation of the K. quasipneumoniae subspecies, between KvMX2/Yh43 and other K. variicola isolates is required to unequivocally qualify these as separate subspecies (27).

Numerous studies have shown that K. pneumoniae has a deep-branching phylogenetic structure with minimal recombination occurring within K. pneumoniae strains and between K. pneumoniae and K. variicola/K. quasipneumoniae (26, 28). Importantly, though, large-scale recombination events may be clinically relevant, as evidenced by research on the origin of the frequently carbapenem-resistant ST258 lineage (29, 30). Our results demonstrate that like K. pneumoniae, K. variicola shows minimal recombination within its genome, and its population structure is composed of numerous clades in a star-like phylogeny. A star-like population structure with deep-branching relationships between isolates (*n* = 29 and *n* = 28) was also found in two previously published K. variicola phylogenetic trees (2, 31).

Similarly to our work, a previous investigation did not identify any geographic distinction when genomes from within the United States were compared to those from outside the United States (2). The 6 genomes in cluster 21 with WUSM_KV_10 were from ICU patient samples in Seattle, WA, which provides the first evidence of clonal groups responsible for K. variicola infections in some settings (32). Although they were closely related compared against all K. variicola genomes, there were still 1,882 SNPs between WUSM_KV_10 and the other 6 genomes. Interestingly, clusters were not restricted to human infections, as cluster 24 contains 3 genomes from bovine mastitis (NL49, NL58, and NL58) and hospital isolates (VRCO0246, VRCO00242, VRCO00244, and VRCO00243) (https://www.ncbi.nlm.nih.gov/bioproject/361595) (33).

As expected for K. variicola, *bla*_LEN_ β-lactamases were the most conserved ARGs. A previous report unexpectedly found a K. variicola isolate that harbored the *bla*_OKP_ gene commonly found in K. quasipneumoniae; however, we did not identify such instances within our cohort (2). Although chromosomally carried in K. pneumoniae, *fosA* was identified in only 1/145 of the K. variicola genomes (34, 35). Additionally, as previously found in K. pneumoniae clinical isolate cohorts, we found *oqxAB* efflux pump genes widespread across K. variicola genomes (36 – 38). Although these genes may be ubiquitous in K. variicola, 0 of 55 isolates we tested had resistance to ciprofloxacin; the single example with intermediate susceptibility carried a *qnrB6* gene. This is not atypical for *Enterobacteriaceae* possessing *oqxAB*, as one study found 100% prevalence of *oxqAB* in K. pneumoniae but no quinolone resistance (37). It is possible that for K. variicola, similarly to K. pneumoniae, high expression of *oqxAB* is essential for phenotypic resistance to quinolones (36). In K. pneumoniae, expansion of clonal groups is associated with carbapenemase carriage (i.e., ST258 and *bla*_KPC_); however, we did not observe any associations between carbapenemase genes and K. variicola clusters. Indeed, only 1.81% (1/55) of K. variicola strains within our institutional cohort had a carbapenemase gene and the regional resistance rate for meropenem between K. pneumoniae and K. variicola in 2017 was similar. *bla*_NDM_-positive K. variicola strains have been identified in clinical and environmental samples, but *bla*_KPC_-positive genomes came exclusively from clinical sources. KPN1481 (*bla*_NDM-1_) was annotated as a urine-derived isolate, but GJ1, GJ2, and GJ3 (all *bla*_NDM-9_) were found in the Gwangju tributary in South Korea (2, 39). In contrast, WUSM_KV_55 (*bla*_KPC-2_) was isolated from bronchoalveolar lavage fluid, KP007 (*bla*_KPC-2_) from an intra-abdominal site, and 223/14 (*bla*_KPC-6_) from a laparotomy wound (40, 41). IncF plasmids, the most abundant replicon identified in the K. variicola cohort, are known carriers of antibiotic resistance genes, including *bla*_CTX-M_ and *bla*_OXA_ β-lactamases (42). Consistent with their widespread identification in K. variicola, IncF plasmids are frequently found in K. pneumoniae and E. coli (43, 44).

K. pneumoniae is the second leading cause of urinary tract infections (45). Given previous misclassification of K. variicola as K. pneumoniae and the high frequency at which K. variicola was isolated from the urinary tract, we were interested in comparing the uropathogenicity of our K. variicola isolates to the well-studied model K. pneumoniae TOP52 isolate (3, 18, 19). We identified strain-dependent virulence capacity, with UTIs from WUSM_KV_39 yielding statistically significant higher bladder CFU than K. pneumoniae TOP52. Quantification of metrics used to study uropathogenicity in E. coli and K. pneumoniae show increased *fimS* in the “ON” orientation and increased FimA production by WUSM_KV_39; these findings provide a plausible explanation for why WUSM_KV_39 performed better than K. pneumoniae TOP52 and all WUSM_KV isolates excluding WUSM_KV_10 (46). While we do not yet understand the role of the putative protein identified between recombinases *fimB* and *fimE* in WUSM_KV_39, one could postulate that this difference may affect fimbrial expression. Additionally, the poorest performer in the urinary tract, WUSM_KV_14, encodes a mutation resulting in a truncated *fimD* usher sequence which likely explains its lack of FimA production. As with other bacterial pathogens, it is likely that specific virulence factors are required for K. variicola competency in distinct body niches (47, 48). Further work is therefore warranted to test if yersiniabactin and allantoin utilization promote lung and liver infections, respectively, in K. variicola as they do in K. pneumoniae (49 – 52).

K. variicola carries usher genes previously identified in K. pneumoniae and 9 novel ushers (53). Interestingly, KveB and KviA are the first report of π usher proteins in *Klebsiella*. The best-studied π operon, *pap* in E. coli, is a major contributor to pyelonephritis as the PapG adhesin can bind Gal-α-(1-4)-Gal exposed on human kidney cells (54). Other usher genes have been shown to be essential for biofilm formation, plant cell adhesion, and murine gut colonization, further demonstrating their role in niche differentiation (53). Clustering of the presence/absence of these ushers showed the absence of KpbC but presence of KvdB in 11 of the WUSM_KV genomes, a phenomenon similar to that observed for UshC and YraJ in E. coli (55). All 4 of these usher types were found in the γ* clade, suggesting an exclusionary form of functional redundancy between usher genes (55). Usher genes and CUP operons are frequently exchanged horizontally between *Enterobacteriaceae* genera (55). Indeed, we have found that the KvhC usher protein has only 76% amino acid identity to any existing proteins in the nonredundant protein sequence database and that the *kvh* operon is situated next to multiple prophage- and transposase-associated genes.

In this investigation, we used phenotypic and genomic analyses to better understand the diversity of K. variicola genomes, both from our institution and across the globe (using publicly available NCBI genomes). Then, we assessed the functional consequences of ARGs and VGs toward antibiotic resistance and uropathogenicity. One limitation of our study is that our mouse infections and phenotypic analyses are performed with nonisogenic strains. If existing genetic modification systems in K. pneumoniae are shown to be useful for gene knockouts in K. variicola, further work can be performed to mechanistically validate our findings. An additional limitation is that ∼30 genomes of K. variicola have been uploaded to NCBI since we initiated our comparison. These may further elucidate differences in population structure, although even with almost 300 genomes, one study indicates that K. pneumoniae diversity remains undersampled (26).

Our work represents the first large-scale genomic analysis of K. variicola across multiple institutions and the first use of a murine model to study K. variicola pathogenesis. We unequivocally show that whole-genome comparisons can separate K. variicola from K. pneumoniae and offer convenient alternative methods for laboratories without access to WGS to differentiate these species. Importantly, we demonstrate that high-risk ARGs and VGs are present in K. variicola genomes from a variety of geographic locations. This may have clinical ramifications, as we demonstrate that some K. variicola clinical isolates can be superior uropathogens compared to K. pneumoniae. Similarly to E. coli and K. pneumoniae, the diversity of CUP operons in these isolates could complement additional acquired virulence genes and enable infection of specific niches. Therefore, it is imperative that K. variicola and K. pneumoniae continue to be differentiated in the clinical laboratory, so that we may apply data on differential gene repertoire, clinical behavior, and niche specificity to the goal of ultimately improving patient outcomes.

## MATERIALS AND METHODS

### Clinical *Klebsiella* collection.

One hundred thirteeen clinical *Klebsiella* species isolates recovered in the Barnes-Jewish Hospital microbiology laboratory (St. Louis, MO) from 2016 to 2017 were evaluated in this study. Of these, 56 were consecutively collected isolates identified by Bruker Biotyper MALDI-TOF MS as K. variicola (research-use-only database v6). This identification was confirmed using a PCR-restriction fragment length polymorphism (RFLP) assay targeting the *yggE* gene (F: 5′-TGTTACTTAAATCGCCCTTACGGG-3′; R: 5′-CAGCGATCTGCAAAACGTCTACT-3′; restriction enzyme: BciVI) that was designed to distinguish K. variicola from K. pneumoniae. A 94.6% proportion (53/56) of isolates were confirmed as K. variicola using the *yggE* PCR-RFLP assay.

The remaining 58 isolates were randomly selected from a banked collection of K. pneumoniae strains historically recovered from clinical specimens (29 from urine, 25 from blood, and 1 each from abdominal wound, tracheal aspirate, bronchial washing, and bile). Each of these isolates underwent Bruker MALDI-TOF MS and *yggE* PCR-RFLP to confirm their identification. Five percent (3/58) were confirmed as K. variicola using MALDI-TOF MS and the *yggE* PCR-RFLP assay.

### Illumina whole-genome sequencing and publicly available *Klebsiella* genomes.

Pure frozen stocks of the presumptive 113 *Klebsiella* isolates were plated on blood agar to isolate single colonies. Approximately 10 colonies were suspended using a sterile cotton swab into water, and total genomic DNA was extracted using the Bacteremia kit (Qiagen). An 0.5-ng amount of DNA was used as input for sequencing libraries using the Nextera kit (Illumina) (56). Libraries were pooled and sequenced on an Illumina NextSeq 2500 high-output system to obtain ∼2.5 million 2 × 150-bp reads. Demultiplexed reads had Illumina adapters removed with Trimmomatic v.36 and decontaminated with DeconSeq v0.4.3 (57, 58). Draft genomes were assembled with SPAdes v3.11.0, and the scaffolds.fasta files were used as input for QUAST v 4.5 to measure the efficacy of assembly (see Table S1 in the supplemental material) (59, 60). All contigs of ≥500 bp in length were annotated for open reading frames with Prokka v1.12 (61).

To increase the number of genomes for downstream analysis, 50 *K. variicola* genomes were obtained from NCBI genomes (https://www.ncbi.nlm.nih.gov/genome/) in September 2017 (Table S1). Additionally, as it is possible that previously sequenced K. variicola may be incorrectly described as K. pneumoniae, we submitted the complete genome of the K. variicola reference strain At-22 to NCBI BLASTN against the nonredundant nucleotide collection and the whole-genome shotgun sequence databases using default settings in September 2017. Using this method, we obtained 41 genomes of K. pneumoniae with the minimum observed query length of 38% at 99% identity (Table S1). Given that the cohort of genomes analyzed in our study includes isolates initially misannotated, we refer to them as either the NCBI genome or assembly (https://www.ncbi.nlm.nih.gov/assembly) accession key. Sequenced and acquired isolates were analyzed using a variety of computational programs (Text S1). *In silico* sequence typing was performed using mlst v2.11 (https://github.com/tseemann/mlst) and the BIGSdb database (https://bigsdb.pasteur.fr/klebsiella/klebsiella.html).

10.1128/mBio.02481-18.1 TEXT S1 Detailed description of the programs used for *in silico* analysis. Download Text S1, DOCX file, 0.04 MB. Copyright © 2018 Potter et al. 2018 Potter et al. This content is distributed under the terms of the Creative Commons Attribution 4.0 International license.

### Antimicrobial susceptibility testing.

K. variicola isolates underwent antimicrobial susceptibility testing per laboratory standard operating procedures using Kirby-Bauer disk diffusion on Mueller-Hinton agar (BD BBL Mueller-Hinton II agar), in accordance with Clinical and Laboratory Standards Institute (CLSI) standards. Disk diffusion results were interpreted using CLSI *Enterobacteriaceae* disk diffusion breakpoints (62). Briefly, 4 to 5 colonies from pure isolates were used to create a 0.5 McFarland suspension of the organism in sterile saline. A sterile, nontoxic cotton swab was dipped into the bacterial suspension, and a lawn of the organism was plated to Mueller-Hinton agar. Antimicrobial Kirby-Bauer disks were applied, and the plate was incubated at 35°C in room air for 16 to 24 h. The diameters of the zones of growth inhibition surrounding each antimicrobial disk were recorded in millimeters.

### Mouse urinary tract infections.

Bacterial strains from our K. variicola cohort and K. pneumoniae TOP52 were used to inoculate 7- to 8-week-old female C3H/HeN mice (Envigo) by transurethral catheterization as previously described (18, 19, 63). The K. variicola strains were selected to encompass a range of genetically distinct isolates. WUSM_KV_03 and WUSM_KV_10 were specifically chosen as they contain the *all* and *ybt* operons, respectively. Static 20-ml cultures were started from freezer stocks, grown in Luria-Bertani (LB) broth at 37°C for 16 h, and centrifuged for 5 min at 8,000 × *g*, and the resultant pellet was resuspended in phosphate-buffered saline (PBS) and diluted to approximately 4 × 10^8^ CFU/ml. Fifty milliliters of this suspension was used to infect each mouse with an inoculum of 2 × 10^7^ CFU/ml. Inocula were verified by serial dilution and plating. At 24 hpi, bladders and kidneys were aseptically harvested, homogenized in sterile PBS via Bullet Blender (Next Advance) for 5 min, serially diluted, and plated on LB agar. All animal procedures were approved by the Institutional Animal Care and Use Committee at Washington University School of Medicine.

### Phase assays.

To determine the orientation of the *fimS* phase switch in *Klebsiella*, a phase assay was adapted as previously described (20). An 817-bp fragment including *fimS* was PCR amplified using *Taq* polymerase (Invitrogen) and the primers 5′-GGGACAGATACGCGTTTGAT-3′ and 5′-GGCCTAACTGAACGGTTTGA-3′ and then digested with HinfI (New England Biolabs). Digestion products were separated by electrophoresis on a 1% agarose gel. A phase-ON switch yields products of 605 and 212 bp, and a phase-OFF switch yields products of 496 and 321 bp.

### FimA and GroEL immunoblots.

Acid-treated, whole-cell immunoblotting was performed as previously described using 1:2,000 rabbit anti-type 1 pilus and 1:500,000 rabbit anti-GroEL (Sigma-Aldrich) primary antibodies (64, 65). Amersham ECL horseradish peroxidase-linked donkey anti-rabbit IgG (GE Healthcare) secondary antibody (1:2,000) was applied, followed by application of Clarity enhanced chemiluminescence (ECL) substrate (Bio-Rad Laboratories). The membrane was developed and imaged using a ChemiDoc MP Imaging System (Bio-Rad Laboratories). Relative band intensities were quantified using Fiji (https://fiji.sc/) (66).

### Statistics.

CFU/bladder and CFU/kidney for both experimental replicates were used as input for ordinary one-way ANOVA to judge significance. Pairwise comparisons of CFU/bladder and CFU/kidney values were performed by using the nonparametric Mann-Whitney U test. Similarly, normalized quantifications of relative FimA amounts (FimA/GroEL) and *fimS* in “ON” position (*fimS* “ON”/*fimS* “OFF”) were compared using the Mann-Whitney U test. All *P* values of <0.05 were considered significant, and all calculations were performed in GraphPad Prism v7.04.

### Accession number(s).

The genomes have all been deposited in NCBI under BioProject accession no. PRJNA473122.

## References

[1] Rosenblueth M, Martinez L, Silva J, Martinez-Romero E 2004 Klebsiella variicola, a novel species with clinical and plant-associated isolates. Syst Appl Microbiol 27:27–35. doi:10.1078/0723-2020-00261. 15053318

[2] Long SW, Linson SE, Ojeda Saavedra M, Cantu C, Davis JJ, Brettin T, Olsen RJ 2017 Whole-genome sequencing of human clinical *Klebsiella pneumoniae* isolates reveals misidentification and misunderstandings of *Klebsiella pneumoniae*, *Klebsiella variicola*, and *Klebsiella quasipneumoniae*. mSphere 2:e00290-17. doi:10.1128/mSphereDirect.00290-17. 28776045 PMC5541162

[3] Berry GJ, Loeffelholz MJ, Williams-Bouyer N 2015 An investigation into laboratory misidentification of a bloodstream *Klebsiella variicola* infection. J Clin Microbiol 53:2793–2794. doi:10.1128/JCM.00841-15. 26063851 PMC4508421

[4] Maatallah M, Vading M, Kabir MH, Bakhrouf A, Kalin M, Naucler P, Brisse S, Giske CG 2014 Klebsiella variicola is a frequent cause of bloodstream infection in the Stockholm area, and associated with higher mortality compared to K. pneumoniae. PLoS One 9:e113539. doi:10.1371/journal.pone.0113539. 25426853 PMC4245126

[5] Andrade BG, de Veiga Ramos N, Marin MF, Fonseca EL, Vicente AC 2014 The genome of a clinical Klebsiella variicola strain reveals virulence-associated traits and a pl9-like plasmid. FEMS Microbiol Lett 360:13–16. doi:10.1111/1574-6968.12583. 25135672

[6] Martínez-Romero E, Rodríguez-Medina N, Beltrán-Rojel M, Toribio-Jiménez J, Garza-Ramos U 2018 Klebsiella variicola and Klebsiella quasipneumoniae with capacity to adapt to clinical and plant settings. Salud Publica Mex 60:29–40. doi:10.21149/8156. 29689654

[7] Martin RM, Bachman MA 2018 Colonization, infection, and the accessory genome of Klebsiella pneumoniae. Front Cell Infect Microbiol 8:4. doi:10.3389/fcimb.2018.00004. 29404282 PMC5786545

[8] Richter M, Rosselló-Móra R 2009 Shifting the genomic gold standard for the prokaryotic species definition. Proc Natl Acad Sci U S A 106:19126–19131. doi:10.1073/pnas.0906412106. 19855009 PMC2776425

[9] Kurtz S, Phillippy A, Delcher AL, Smoot M, Shumway M, Antonescu C, Salzberg SL 2004 Versatile and open software for comparing large genomes. Genome Biol 5:R12. doi:10.1186/gb-2004-5-2-r12. 14759262 PMC395750

[10] Richter M, Rosselló-Móra R, Oliver Glöckner F, Peplies J 2016 JSpeciesWS: a web server for prokaryotic species circumscription based on pairwise genome comparison. Bioinformatics 32:929–931. doi:10.1093/bioinformatics/btv681. 26576653 PMC5939971

[11] Long SW, Linson SE, Ojeda Saavedra M, Cantu C, Davis JJ, Brettin T, Olsen RJ 2017 Whole-genome sequencing of a human clinical isolate of the novel species *Klebsiella quasivariicola* sp. nov. Genome Announc 5:e01057-17. doi:10.1128/genomeA.01057-17. 29051239 PMC5646392

[12] Mostowy R, Croucher NJ, Andam CP, Corander J, Hanage WP, Marttinen P 2017 Efficient inference of recent and ancestral recombination within bacterial populations. Mol Biol Evol 34:1167–1182. doi:10.1093/molbev/msx066. 28199698 PMC5400400

[13] Cheng L, Connor TR, Siren J, Aanensen DM, Corander J 2013 Hierarchical and spatially explicit clustering of DNA sequences with BAPS software. Mol Biol Evol 30:1224–1228. doi:10.1093/molbev/mst028. 23408797 PMC3670731

[14] Treangen TJ, Ondov BD, Koren S, Phillippy AM 2014 The Harvest suite for rapid core-genome alignment and visualization of thousands of intraspecific microbial genomes. Genome Biol 15:524. doi:10.1186/s13059-014-0524-x. 25410596 PMC4262987

[15] Rose R, Lamers SL, Dollar JJ, Grabowski MK, Hodcroft EB, Ragonnet-Cronin M, Wertheim JO, Redd AD, German D, Laeyendecker O 2017 Identifying transmission clusters with Cluster Picker and HIV-TRACE. AIDS Res Hum Retroviruses 33:211–218. doi:10.1089/AID.2016.0205. 27824249 PMC5333565

[16] Kleinheinz KA, Joensen KG, Larsen MV 2014 Applying the ResFinder and VirulenceFinder web-services for easy identification of acquired antibiotic resistance and E. coli virulence genes in bacteriophage and prophage nucleotide sequences. Bacteriophage 4:e27943. doi:10.4161/bact.27943. 24575358 PMC3926868

[17] Carattoli A, Zankari E, García-Fernández A, Voldby Larsen M, Lund O, Villa L, Møller Aarestrup F, Hasman H 2014 In silico detection and typing of plasmids using PlasmidFinder and plasmid multilocus sequence typing. Antimicrob Agents Chemother 58:3895–3903. doi:10.1128/AAC.02412-14. 24777092 PMC4068535

[18] Johnson JG, Spurbeck RR, Sandhu SK, Matson JS 2014 Genome sequence of *Klebsiella pneumoniae* urinary tract isolate Top52. Genome Announc 2:e00668-14. doi:10.1128/genomeA.00668-14. 24994806 PMC4082006

[19] Rosen DA, Pinkner JS, Jones JM, Walker JN, Clegg S, Hultgren SJ 2008 Utilization of an intracellular bacterial community pathway in *Klebsiella pneumoniae* urinary tract infection and the effects of FimK on type 1 pilus expression. Infect Immun 76:3337–3345. doi:10.1128/IAI.00090-08. 18411285 PMC2446714

[20] Struve C, Bojer M, Krogfelt KA 2008 Characterization of *Klebsiella pneumoniae* type 1 fimbriae by detection of phase variation during colonization and infection and impact on virulence. Infect Immun 76:4055–4065. doi:10.1128/IAI.00494-08. 18559432 PMC2519443

[21] Abraham JM, Freitag CS, Clements JR, Eisenstein BI 1985 An invertible element of DNA controls phase variation of type 1 fimbriae of Escherichia coli. Proc Natl Acad Sci U S A 82:5724–5727. doi:10.1073/pnas.82.17.5724. 2863818 PMC390624

[22] Sullivan MJ, Petty NK, Beatson SA 2011 Easyfig: a genome comparison visualizer. Bioinformatics 27:1009–1010. doi:10.1093/bioinformatics/btr039. 21278367 PMC3065679

[23] Waterhouse AM, Procter JB, Martin DM, Clamp M, Barton GJ 2009 Jalview version 2—a multiple sequence alignment editor and analysis workbench. Bioinformatics 25:1189–1191. doi:10.1093/bioinformatics/btp033. 19151095 PMC2672624

[24] Busch A, Waksman G 2012 Chaperone-usher pathways: diversity and pilus assembly mechanism. Philos Trans R Soc Lond B Biol Sci 367:1112–1122. doi:10.1098/rstb.2011.0206. 22411982 PMC3297437

[25] Nuccio SP, Baumler AJ 2007 Evolution of the chaperone/usher assembly pathway: fimbrial classification goes Greek. Microbiol Mol Biol Rev 71:551–575. doi:10.1128/MMBR.00014-07. 18063717 PMC2168650

[26] Holt KE, Wertheim H, Zadoks RN, Baker S, Whitehouse CA, Dance D, Jenney A, Connor TR, Hsu LY, Severin J, Brisse S, Cao H, Wilksch J, Gorrie C, Schultz MB, Edwards DJ, Nguyen KV, Nguyen TV, Dao TT, Mensink M, Minh VL, Nhu NT, Schultsz C, Kuntaman K, Newton PN, Moore CE, Strugnell RA, Thomson NR 2015 Genomic analysis of diversity, population structure, virulence, and antimicrobial resistance in Klebsiella pneumoniae, an urgent threat to public health. Proc Natl Acad Sci U S A 112:E3574–E3581. doi:10.1073/pnas.1501049112. 26100894 PMC4500264

[27] Brisse S, Passet V, Grimont PA 2014 Description of Klebsiella quasipneumoniae sp. nov., isolated from human infections, with two subspecies, Klebsiella quasipneumoniae subsp. quasipneumoniae subsp. nov. and Klebsiella quasipneumoniae subsp. similipneumoniae subsp. nov., and demonstration that Klebsiella singaporensis is a junior heterotypic synonym of Klebsiella variicola. Int J Syst Evol Microbiol 64:3146–3152. doi:10.1099/ijs.0.062737-0. 24958762

[28] Moradigaravand D, Martin V, Peacock SJ, Parkhill J 2017 Evolution and epidemiology of multidrug-resistant *Klebsiella pneumoniae* in the United Kingdom and Ireland. mBio 8:e01976-16. doi:10.1128/mBio.01976-16. 28223459 PMC5358916

[29] Wyres KL, Gorrie C, Edwards DJ, Wertheim HF, Hsu LY, Van Kinh N, Zadoks R, Baker S, Holt KE 2015 Extensive capsule locus variation and large-scale genomic recombination within the Klebsiella pneumoniae clonal group 258. Genome Biol Evol 7:1267–1279. doi:10.1093/gbe/evv062. 25861820 PMC4453057

[30] Chen L, Mathema B, Pitout JD, DeLeo FR, Kreiswirth BN 2014 Epidemic *Klebsiella pneumoniae* ST258 is a hybrid strain. mBio 5:e01355-14. doi:10.1128/mBio.01355-14. 24961694 PMC4073492

[31] Gorrie CL, Mirceta M, Wick RR, Edwards DJ, Thomson NR, Strugnell RA, Pratt NF, Garlick JS, Watson KM, Pilcher DV, McGloughlin SA, Spelman DW, Jenney AWJ, Holt KE 2017 Gastrointestinal carriage is a major reservoir of Klebsiella pneumoniae infection in intensive care patients. Clin Infect Dis 65:208–215. doi:10.1093/cid/cix270. 28369261 PMC5850561

[32] Roach DJ, Burton JN, Lee C, Stackhouse B, Butler-Wu SM, Cookson BT, Shendure J, Salipante SJ 2015 A year of infection in the intensive care unit: prospective whole genome sequencing of bacterial clinical isolates reveals cryptic transmissions and novel microbiota. PLoS Genet 11:e1005413. doi:10.1371/journal.pgen.1005413. 26230489 PMC4521703

[33] Davidson FW, Whitney HG, Tahlan K 2015 Genome sequences of *Klebsiella variicola* isolates from dairy animals with bovine mastitis from Newfoundland, Canada. Genome Announc 3:e00938-15. doi:10.1128/genomeA.00938-15. 26358587 PMC4566169

[34] Guo Q, Tomich AD, McElheny CL, Cooper VS, Stoesser N, Wang M, Sluis-Cremer N, Doi Y 2016 Glutathione-S-transferase FosA6 of Klebsiella pneumoniae origin conferring fosfomycin resistance in ESBL-producing Escherichia coli. J Antimicrob Chemother 71:2460–2465. doi:10.1093/jac/dkw177. 27261267 PMC4992852

[35] Ito R, Mustapha MM, Tomich AD, Callaghan JD, McElheny CL, Mettus RT, Shanks RMQ, Sluis-Cremer N, Doi Y 2017 Widespread fosfomycin resistance in Gram-negative bacteria attributable to the chromosomal *fosA* gene. mBio 8:e00749-17. doi:10.1128/mBio.00749-17. 28851843 PMC5574708

[36] Rodríguez-Martínez JM, Díaz de Alba P, Briales A, Machuca J, Lossa M, Fernández-Cuenca F, Rodríguez Baño J, Martínez-Martínez L, Pascual Á 2013 Contribution of OqxAB efflux pumps to quinolone resistance in extended-spectrum-beta-lactamase-producing Klebsiella pneumoniae. J Antimicrob Chemother 68:68–73. doi:10.1093/jac/dks377. 23011289

[37] Perez F, Rudin SD, Marshall SH, Coakley P, Chen L, Kreiswirth BN, Rather PN, Hujer AM, Toltzis P, van Duin D, Paterson DL, Bonomo RA 2013 OqxAB, a quinolone and olaquindox efflux pump, is widely distributed among multidrug-resistant *Klebsiella pneumoniae* isolates of human origin. Antimicrob Agents Chemother 57:4602–4603. doi:10.1128/AAC.00725-13. 23817374 PMC3754307

[38] Yuan J, Xu X, Guo Q, Zhao X, Ye X, Guo Y, Wang M 2012 Prevalence of the oqxAB gene complex in Klebsiella pneumoniae and Escherichia coli clinical isolates. J Antimicrob Chemother 67:1655–1659. doi:10.1093/jac/dks086. 22438434

[39] Di DY, Jang J, Unno T, Hur HG 2017 Emergence of Klebsiella variicola positive for NDM-9, a variant of New Delhi metallo-beta-lactamase, in an urban river in South Korea. J Antimicrob Chemother 72:1063–1067. doi:10.1093/jac/dkw547. 28087584

[40] Cienfuegos-Gallet AV, Chen L, Kreiswirth BN, Jimenez JN 2017 Colistin resistance in carbapenem-resistant *Klebsiella pneumoniae* mediated by chromosomal integration of plasmid DNA. Antimicrob Agents Chemother 61:e00404-17. doi:10.1128/AAC.00404-17. 28507118 PMC5527652

[41] Ahmad N, Chong TM, Hashim R, Shukor S, Yin WF, Chan KG 2015 Draft genome of multidrug-resistant Klebsiella pneumoniae 223/14 carrying KPC-6, isolated from a general hospital in Malaysia. J Genomics 3:97–98. doi:10.7150/jgen.13910. 26816553 PMC4716803

[42] Carattoli A 2009 Resistance plasmid families in *Enterobacteriaceae*. Antimicrob Agents Chemother 53:2227–2238. doi:10.1128/AAC.01707-08. 19307361 PMC2687249

[43] Dolejska M, Villa L, Dobiasova H, Fortini D, Feudi C, Carattoli A 2013 Plasmid content of a clinically relevant *Klebsiella pneumoniae* clone from the Czech Republic producing CTX-M-15 and QnrB1. Antimicrob Agents Chemother 57:1073–1076. doi:10.1128/AAC.01886-12. 23229477 PMC3553734

[44] Shin J, Choi MJ, Ko KS 2012 Replicon sequence typing of IncF plasmids and the genetic environments of blaCTX-M-15 indicate multiple acquisitions of blaCTX-M-15 in Escherichia coli and Klebsiella pneumoniae isolates from South Korea. J Antimicrob Chemother 67:1853–1857. doi:10.1093/jac/dks143. 22566590

[45] Flores-Mireles AL, Walker JN, Caparon M, Hultgren SJ 2015 Urinary tract infections: epidemiology, mechanisms of infection and treatment options. Nat Rev Microbiol 13:269–284. doi:10.1038/nrmicro3432. 25853778 PMC4457377

[46] Schwan WR, Ding H 2017 Temporal regulation of fim genes in uropathogenic Escherichia coli during infection of the murine urinary tract. J Pathog 2017:8694356. doi:10.1155/2017/8694356. 29445547 PMC5763102

[47] Chmiela M, Miszczyk E, Rudnicka K 2014 Structural modifications of Helicobacter pylori lipopolysaccharide: an idea for how to live in peace. World J Gastroenterol 20:9882–9897. doi:10.3748/wjg.v20.i29.9882. 25110419 PMC4123370

[48] Hill C 2012 Virulence or niche factors: what’s in a name? J Bacteriol 194:5725–5727. doi:10.1128/JB.00980-12. 22821969 PMC3486107

[49] Lawlor MS, O’Connor C, Miller VL 2007 Yersiniabactin is a virulence factor for *Klebsiella pneumoniae* during pulmonary infection. Infect Immun 75:1463–1472. doi:10.1128/IAI.00372-06. 17220312 PMC1828572

[50] Bachman MA, Oyler JE, Burns SH, Caza M, Lepine F, Dozois CM, Weiser JN 2011 *Klebsiella pneumoniae* yersiniabactin promotes respiratory tract infection through evasion of lipocalin 2. Infect Immun 79:3309–3316. doi:10.1128/IAI.05114-11. 21576334 PMC3147564

[51] Chou HC, Lee CZ, Ma LC, Fang CT, Chang SC, Wang JT 2004 Isolation of a chromosomal region of *Klebsiella pneumoniae* associated with allantoin metabolism and liver infection. Infect Immun 72:3783–3792. doi:10.1128/IAI.72.7.3783-3792.2004. 15213119 PMC427404

[52] Compain F, Babosan A, Brisse S, Genel N, Audo J, Ailloud F, Kassis-Chikhani N, Arlet G, Decré D 2014 Multiplex PCR for detection of seven virulence factors and K1/K2 capsular serotypes of *Klebsiella pneumoniae*. J Clin Microbiol 52:4377–4380. doi:10.1128/JCM.02316-14. 25275000 PMC4313302

[53] Khater F, Balestrino D, Charbonnel N, Dufayard JF, Brisse S, Forestier C 2015 In silico analysis of usher encoding genes in Klebsiella pneumoniae and characterization of their role in adhesion and colonization. PLoS One 10:e0116215. doi:10.1371/journal.pone.0116215. 25751658 PMC4353729

[54] Verger D, Bullitt E, Hultgren SJ, Waksman G 2007 Crystal structure of the P pilus rod subunit PapA. PLoS Pathog 3:e73. doi:10.1371/journal.ppat.0030073. 17511517 PMC1868955

[55] Stubenrauch CJ, Dougan G, Lithgow T, Heinz E 2017 Constraints on lateral gene transfer in promoting fimbrial usher protein diversity and function. Open Biol 7:170144. doi:10.1098/rsob.170144. 29142104 PMC5717340

[56] Baym M, Kryazhimskiy S, Lieberman TD, Chung H, Desai MM, Kishony R 2015 Inexpensive multiplexed library preparation for megabase-sized genomes. PLoS One 10:e0128036. doi:10.1371/journal.pone.0128036. 26000737 PMC4441430

[57] Bolger AM, Lohse M, Usadel B 2014 Trimmomatic: a flexible trimmer for Illumina sequence data. Bioinformatics 30:2114–2120. doi:10.1093/bioinformatics/btu170. 24695404 PMC4103590

[58] Schmieder R, Edwards R 2011 Fast identification and removal of sequence contamination from genomic and metagenomic datasets. PLoS One 6:e17288. doi:10.1371/journal.pone.0017288. 21408061 PMC3052304

[59] Bankevich A, Nurk S, Antipov D, Gurevich AA, Dvorkin M, Kulikov AS, Lesin VM, Nikolenko SI, Pham S, Prjibelski AD, Pyshkin AV, Sirotkin AV, Vyahhi N, Tesler G, Alekseyev MA, Pevzner PA 2012 SPAdes: a new genome assembly algorithm and its applications to single-cell sequencing. J Comput Biol 19:455–477. doi:10.1089/cmb.2012.0021. 22506599 PMC3342519

[60] Gurevich A, Saveliev V, Vyahhi N, Tesler G 2013 QUAST: quality assessment tool for genome assemblies. Bioinformatics 29:1072–1075. doi:10.1093/bioinformatics/btt086. 23422339 PMC3624806

[61] Seemann T 2014 Prokka: rapid prokaryotic genome annotation. Bioinformatics 30:2068–2069. doi:10.1093/bioinformatics/btu153. 24642063

[62] Clinical and Laboratory Standards Institute. 2017 Performance standards for antimicrobial susceptibility testing, 27th ed. CLSI supplement M100. Clinical and Laboratory Standards Institute, Wayne, PA.

[63] Mulvey MA, Lopez-Boado YS, Wilson CL, Roth R, Parks WC, Heuser J, Hultgren SJ 1998 Induction and evasion of host defenses by type 1-piliated uropathogenic Escherichia coli. Science 282:1494–1497. doi:10.1126/science.282.5393.1494. 9822381

[64] Garofalo CK, Hooton TM, Martin SM, Stamm WE, Palermo JJ, Gordon JI, Hultgren SJ 2007 *Escherichia coli* from urine of female patients with urinary tract infections is competent for intracellular bacterial community formation. Infect Immun 75:52–60. doi:10.1128/IAI.01123-06. 17074856 PMC1828379

[65] Pinkner JS, Remaut H, Buelens F, Miller E, Aberg V, Pemberton N, Hedenstrom M, Larsson A, Seed P, Waksman G, Hultgren SJ, Almqvist F 2006 Rationally designed small compounds inhibit pilus biogenesis in uropathogenic bacteria. Proc Natl Acad Sci U S A 103:17897–17902. doi:10.1073/pnas.0606795103. 17098869 PMC1693844

[66] Schindelin J, Arganda-Carreras I, Frise E, Kaynig V, Longair M, Pietzsch T, Preibisch S, Rueden C, Saalfeld S, Schmid B, Tinevez JY, White DJ, Hartenstein V, Eliceiri K, Tomancak P, Cardona A 2012 Fiji: an open-source platform for biological-image analysis. Nat Methods 9:676–682. doi:10.1038/nmeth.2019. 22743772 PMC3855844

