# Supplementary figures and images for: Population Structure, Antibiotic Resistance, and Uropathogenicity of Klebsiella variicola
Source: mBio. 2018 Dec 18;9(6):e02481-18. doi: 10.1128/mBio.02481-18 (PMC6299229; doi:10.1128/mBio.02481-18)

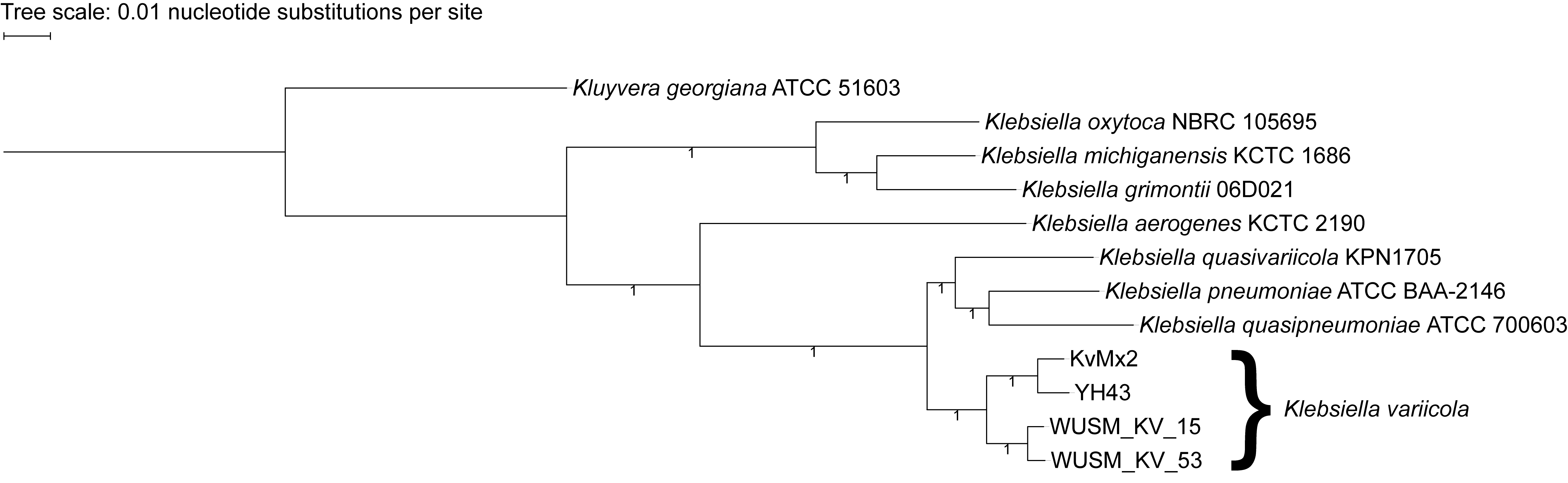

Supplement: FIG S1 [file mbo006184237sf1.tif]

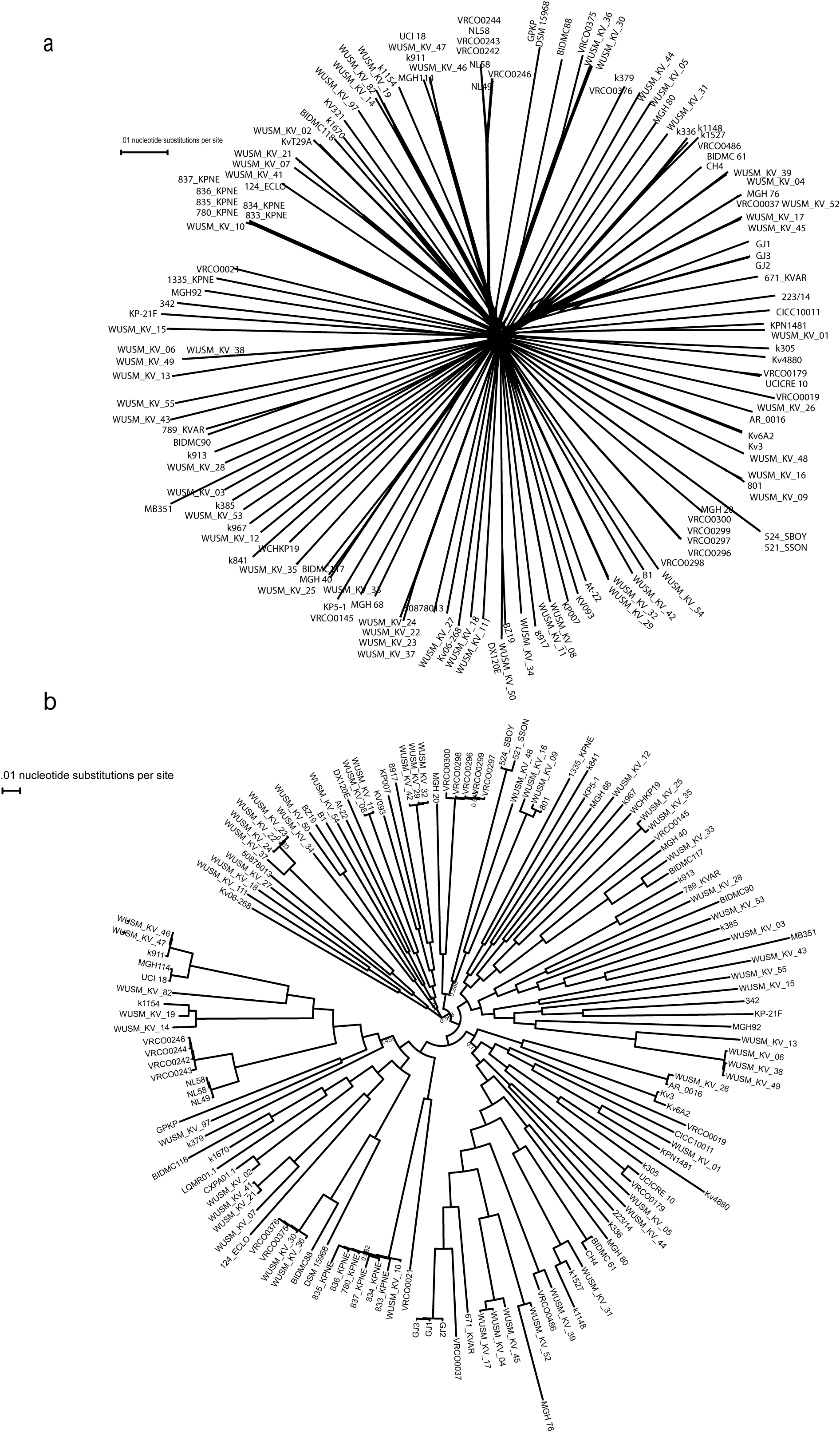

Supplement: FIG S2 [file mbo006184237sf2.tif]

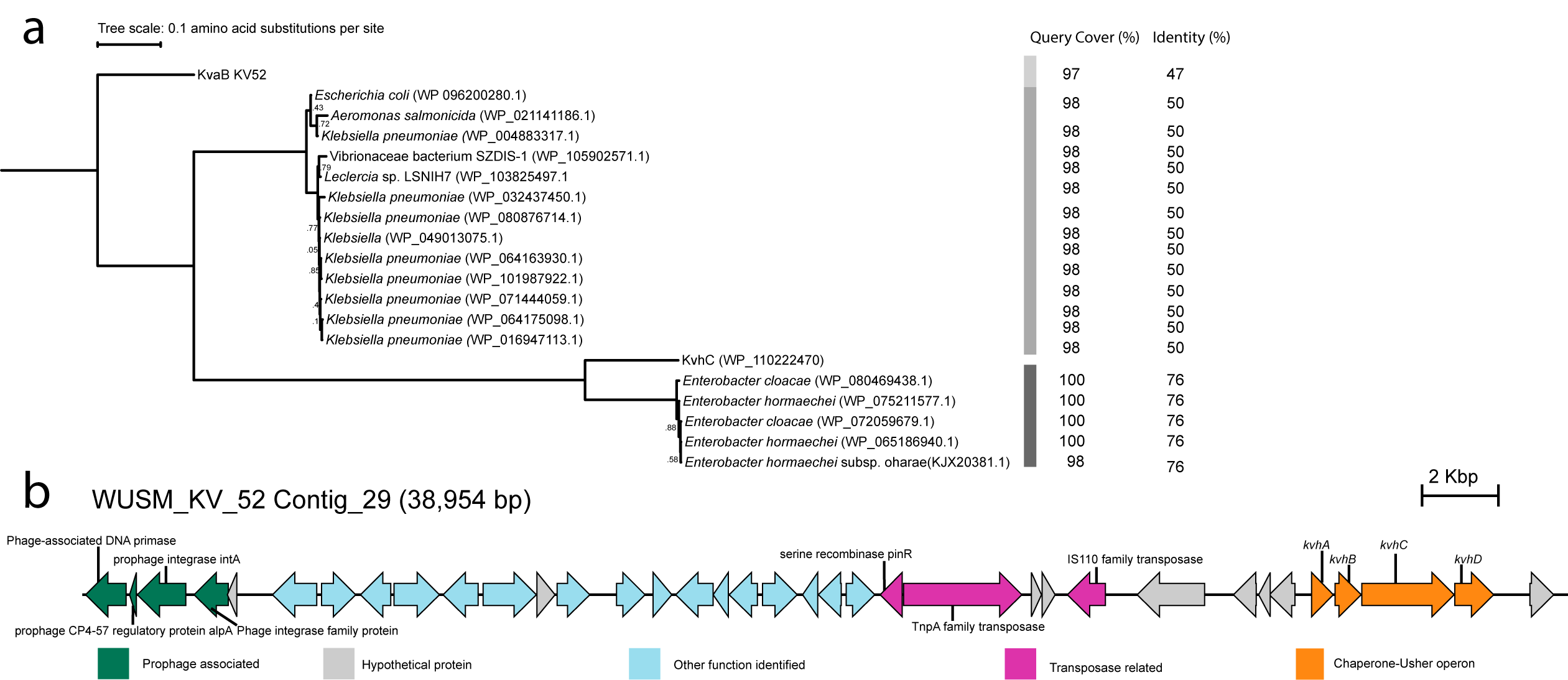

Supplement: FIG S3 [file mbo006184237sf3.tif]
