# Supplementary material for: Population Structure, Antibiotic Resistance, and Uropathogenicity of Klebsiella variicola
Source: mBio. 2018 Dec 18;9(6):e02481-18. doi: 10.1128/mBio.02481-18 (PMC6299229; doi:10.1128/mBio.02481-18)
Supplement: TEXT S1 [file mbo006184237s1.docx]

**Average Nucleotide Identity**

The .fsa files from prokka, containing all contigs > 500 bp in length from our *Klebsiella* cohort, were added to a separate directory with the publicly available suspected *K. variicola* sequences and the genomes for *K. pneumoniae* CAV1042, *K. pneumoniae* HS11286, and *K. quasipneumoniae* ATCC 7000603. We used the command line ANI tool pyANI (https://github.com/widdowquinn/pyani) with the mummer method to compute the pairwise average nucleotide identity among the 207 genomes. The resulting ANIm matrix was viewed as a clustermap using seaborn (https://seaborn.pydata.org/index.html).

To further confirm the delineation of *K. variicola* into a major and minor clade, we repeated the ANI analysis using the Jspecies webserver (<http://jspecies.ribohost.com/jspeciesws/#analyse>) BLAST method in April 2018 between KvMX2 and Yh43 (1) (Table S3).

**Core-genome alignment and visualizations**

Analysis of K. variicola in relation to other species in the Klebsiella genus was performed by running roary v 3.8.0 with 90% identity on .gff output of prokka from 4 *K. variicola* strains and a strain from *K. pneumoniae*, *K. quasipnuemoniae*, *K. quasivariicola*, *K. grimontii*, *K. aerogenes*, *K. oxytoca*, and *K. michiganensis*, with *Kluyvera georgiana* used as an outgroup. The 1,262 genes were aligned in roary with PRANK v1.0 and convereted into a newick file with FastTree v2.1.10 (Table S4).

Initial delineation of the population structure for *K. variicola* was performed by moving the .gff file output from prokka into a separate directory and using roary v3.8.0 to identify the core-genomes (those shared by 100% of all genomes) and PRANK v1.0 to align them (Table S5) (2, 3). The resulting alignment file was constructed into a newick tree using FastTree v2.1.10 and viewed in ITOL (4, 5). To quantify recombination between the strains and identify lineages in a phylogenetic independent manner, we performed FastGear on the roary alignment file of the 3,430 genes shared by the 145 *K. variicola* genomes (Table S6).

To understand the population structure of the 143 genomes in the second lineage, we used parSNP within the harvest suite to construct phylogenetic trees from the scaffolds.fasta file with and without recombination (6) (Fig. S1) (Table S7). As an orthologous method, we created an approximate-maximum-likelihood tree of 3,500 core-genes shared by the 143 *K. variicola* isolates in the second lineage from roary and FastTree (Table S8) (Fig. S2). The resulting newick tree and alignment file were used to identify clusters of isolates with ClusterPicker v1.3 (7). Clusters were identified using an initial and main support threshold of .9, a genetic distance threshold of 4.5, and large cluster threshold of 10. The clusters identified were then visualized on the parSNP tree without recombination with 100% concordance (26/26 clusters). To alternatively view the population structure of *K. variicola*, just the SNP locations were identified by performing snp-sites on the roary alignment file of the 143 genomes in the major clade (8). This file was visualized as an unrooted equal angle Nearest Neighbor phylogenetic network in SplitsTrees v4 (9, 10). To improve the resolution on the highly related isolates in cluster 21, which contained WUSM_KV_10 and 6 isolates from an investigation of infectious agents in an ICU, we used roary v.3.8.0 to identify the 4,867 core-genes for these 7 genomes at 95% identity{26230489}(Table S9). Single Nucleotide Polymorphisms in these core-genes were identified using SNP-sites{28348851}(Table S10).

A final phylogenetic tree was created by performing roary and PRANK on *Klebsiella aerogenes* KCTC 2190*, K. quasipneumoniae* ATCC 700603, and *K. pneumoniae* ATCC BAA-1705 at >90% identity. The 2,932 genes shared by all isolates were used to construct a newick file using FastTree (Table S11).

**Antibiotic Resistance Gene, Virulence Gene identification**

Acquired ARGs were identified from the .ffn output of prokka using the command line version of ResFinder with default parameters against all available database classes (11). Similarity, the plasmid replicons were identified using the command line version of PlasmidFinder against the Enterobacteriaceae database{24777092}. The number of isolates with > or <= the median number of ARGs and plasmid replicons were tallied and used as input for a Chi-Square test calculator (<https://www.socscistatistics.com/tests/chisquare2/Default2.aspx>) (Table S12). Virulence genes were annotated by downloading the BIGSDB (http://bigsdb.pasteur.fr/klebsiella/klebsiella.html) list of virulence genes in January 2018 and making them into a custom blast nucleotide database. BLASTN was used to query the .ffn output of prokka against the virulence gene database, with hits requiring 95% identity (Table S12).

Acquired ARGs in the WUSM *K. variicola* cohort were viewed as a network diagram in Cytoscape v 3.4.0 by constructing a text file where each source node is represented by a unique ARG and the target is the isolate genome with an edge weight of 1 (12) (Table S12).

***fim* operon visualization**

The complete *fim* operon sequence was obtained from the draft genome of TOP52, a model uropathogenic *K. pneumoniae* strain (13, 14). BLASTN was used to extract the *fim* operon containing contigs from the draft genomes of the strains used for mouse infections and *in vitro* experiments. The contigs were reannotated using prokka, and the GenBank files were visualized in EasyFig without any pairwise BLAST identity values (15). ORFs were colorized based on suspected function. To visualize any SNPs between the different operons, the complete sequence containing the operon was aligned using MUSCLE and visualized in JALView (16).

**Usher analysis**

Putative usher sequenced were obtained from the pan-genome of the WUSM *K. variicola* isolates by searching the gene_presence_absence.csv output of roary for genes or annotations containing the phrase “outer membrane usher”. To ensure that all possible ORFs were identified, the pan_genome_refence.fa containing representatives of every gene in the pan-genome, was compared against the *fimD* usher nucleotide sequencing using the BLASTN webserver in April 2018. To determinate if any *K. variicola* usher sequences were already described, we used protein BLAST to compare the amino acid sequences against *fim, mrk, kpa, kpb, kpc, kpd, kpe, kpf, kpg,* and *kpj* (17, 18) The amino acid sequence for every usher sequence was obtained and added into a multifasta containing representative usher sequences from various Gram-negative phyla described by Nuccio and Baumler (19) (Table S13). The multifasta was aligned using MUSCLE and then converted into a newick tree using FastTree (20, 21). The resulting tree was viewed in ITOL had clades annotated from the Nuccio and Baumler scheme and terminal branches from *K. variicola* labeled by the operon name.

The distribution of all usher sequences in the WUSM *K. variicola* pan-genome was surveyed by creating a presence/absence matrix for all usher genes and then hierarchically clustering the matrix in Seaborn. The resulting heatmap was annotated by name of operon, name of isolate, and conservation within *K. variicola*. Suspected sequences with truncated ushers were inspected when prokka annotation identified two adjacent usher ORFs and manually annotated on the heatmap.

The *K. variicola* specific usher sequences identified were submitted against the nonredundant protein sequences database in April 2018 and had the top hit blast identity values recorded (Table S14). Given that KvhC had the lowest amino acid percent identity of the newly characterized usher proteins, we obtained all the amino acid sequences for blast hits greater than 49% identity and 99% the query length to construct a phylogenetic tree. The amino acid sequences were aligned using MUSCLE and rooted to the nearest usher sequence in our collection, KvaC. The alignment file was made into a newick tree using FastTree and then viewed in ITOL with percent identity and query length values added to each node.

The contig containing the *kvh* operon was extracted from the genome of strain WUSM_KV_52 and reannotated with prokka. The resulting GenBank file was viewed in easyfig to observe genes syntenic with the operon. All ORFs were submitted to BLASTP in April 2018 against the nonredundant protein database to identity putative functions. ORFs with suspected roles in transposase and prophage activity were specifically marked.

1. Richter M, Rossello-Mora R, Oliver Glockner F, Peplies J. 2016. JSpeciesWS: a web server for prokaryotic species circumscription based on pairwise genome comparison. Bioinformatics 32:929-31.

2. Page AJ, Cummins CA, Hunt M, Wong VK, Reuter S, Holden MT, Fookes M, Falush D, Keane JA, Parkhill J. 2015. Roary: rapid large-scale prokaryote pan genome analysis. Bioinformatics 31:3691-3.

3. Loytynoja A. 2014. Phylogeny-aware alignment with PRANK. Methods Mol Biol 1079:155-70.

4. Price MN, Dehal PS, Arkin AP. 2010. FastTree 2--approximately maximum-likelihood trees for large alignments. PLoS One 5:e9490.

5. Letunic I, Bork P. 2016. Interactive tree of life (iTOL) v3: an online tool for the display and annotation of phylogenetic and other trees. Nucleic Acids Res 44:W242-5.

6. Treangen TJ, Ondov BD, Koren S, Phillippy AM. 2014. The Harvest suite for rapid core-genome alignment and visualization of thousands of intraspecific microbial genomes. Genome Biol 15:524.

7. Rose R, Lamers SL, Dollar JJ, Grabowski MK, Hodcroft EB, Ragonnet-Cronin M, Wertheim JO, Redd AD, German D, Laeyendecker O. 2017. Identifying Transmission Clusters with Cluster Picker and HIV-TRACE. AIDS Res Hum Retroviruses 33:211-218.

8. Page AJ, Taylor B, Delaney AJ, Soares J, Seemann T, Keane JA, Harris SR. 2016. SNP-sites: rapid efficient extraction of SNPs from multi-FASTA alignments. Microb Genom 2:e000056.

9. Huson DH, Bryant D. 2006. Application of phylogenetic networks in evolutionary studies. Mol Biol Evol 23:254-67.

10. Nascimento M, Sousa A, Ramirez M, Francisco AP, Carrico JA, Vaz C. 2017. PHYLOViZ 2.0: providing scalable data integration and visualization for multiple phylogenetic inference methods. Bioinformatics 33:128-129.

11. Kleinheinz KA, Joensen KG, Larsen MV. 2014. Applying the ResFinder and VirulenceFinder web-services for easy identification of acquired antibiotic resistance and E. coli virulence genes in bacteriophage and prophage nucleotide sequences. Bacteriophage 4:e27943.

12. Shannon P, Markiel A, Ozier O, Baliga NS, Wang JT, Ramage D, Amin N, Schwikowski B, Ideker T. 2003. Cytoscape: a software environment for integrated models of biomolecular interaction networks. Genome Res 13:2498-504.

13. Johnson JG, Spurbeck RR, Sandhu SK, Matson JS. 2014. Genome Sequence of Klebsiella pneumoniae Urinary Tract Isolate Top52. Genome Announc 2.

14. Rosen DA, Pinkner JS, Jones JM, Walker JN, Clegg S, Hultgren SJ. 2008. Utilization of an intracellular bacterial community pathway in Klebsiella pneumoniae urinary tract infection and the effects of FimK on type 1 pilus expression. Infect Immun 76:3337-45.

15. Sullivan MJ, Petty NK, Beatson SA. 2011. Easyfig: a genome comparison visualizer. Bioinformatics 27:1009-10.

16. Waterhouse AM, Procter JB, Martin DM, Clamp M, Barton GJ. 2009. Jalview Version 2--a multiple sequence alignment editor and analysis workbench. Bioinformatics 25:1189-91.

17. Khater F, Balestrino D, Charbonnel N, Dufayard JF, Brisse S, Forestier C. 2015. In silico analysis of usher encoding genes in Klebsiella pneumoniae and characterization of their role in adhesion and colonization. PLoS One 10:e0116215.

18. Wu CC, Huang YJ, Fung CP, Peng HL. 2010. Regulation of the Klebsiella pneumoniae Kpc fimbriae by the site-specific recombinase KpcI. Microbiology 156:1983-92.

19. Nuccio SP, Baumler AJ. 2007. Evolution of the chaperone/usher assembly pathway: fimbrial classification goes Greek. Microbiol Mol Biol Rev 71:551-75.

20. Edgar RC. 2004. MUSCLE: multiple sequence alignment with high accuracy and high throughput. Nucleic Acids Res 32:1792-7.

21. Price MN, Dehal PS, Arkin AP. 2009. FastTree: computing large minimum evolution trees with profiles instead of a distance matrix. Mol Biol Evol 26:1641-50.
